# Olive Waste as Feed Supplement: Morphometrical Effects on the *Ansa spiralis coli* in Lambs

**DOI:** 10.1111/ahe.70114

**Published:** 2026-04-24

**Authors:** Gabriel Soares Tosatto Pellenz, Gabriel Navarrina Soares, Marcelo José Böck, Vitor Pires Pereira, William Soares Teixeira, Sérgio Carvalho, Sokol Duro, William Pérez, Luciano de Morais‐Pinto

**Affiliations:** ^1^ Laboratório de Design Anatômico/LabDA‐Departamento de Morfologia Universidade Federal de Santa Maria Rio Grande do Sul Brazil; ^2^ Laboratório de Ovinocultura‐Departamento de Zootecnia Universidade Federal de Santa Maria Rio Grande do Sul Brazil; ^3^ Department of Morphofunctional Modules, Faculty of Veterinary Medicine Agricultural University Tirana Albania; ^4^ National Research System and PEDECIBA Montevideo Uruguay

**Keywords:** animal nutrition, digestive system, environment pollution, olive waste, small ruminant

## Abstract

Feedlot systems for small ruminants require nutritionally adequate and viable diets, and the incorporation of olive residues can offer a sustainable and economically advantageous alternative. This study evaluated the effects of including olive waste at 7.5%, 15%, 22.5% and 30% in the diets of Texel × Ile‐de‐France lambs, assessing the architecture and structure of the *Ansa spiralis coli* through gross anatomy, morphometry and light microscopy using ImageJ software (Fiji). Diets with up to 22.5% olive residue did not modify the morphology or morphometric parameters of the *Ansa spiralis coli*, with all specimens exhibiting three centripetal and three‐and‐a‐half centrifugal gyri and a mean length ratio of 1.18; however, the 30% inclusion level significantly increased the thickness of the tunica muscularis, suggesting potential effects on intestinal motility. These findings indicate that up to 22.5% olive residue can be safely incorporated into lamb feed as a sustainable and economically viable option, helping bridge knowledge gaps on olive byproduct use in ruminant nutrition while offering practical solutions for producers seeking to optimize regional feed resources and maintain animal health and productivity.

## 1 Introduction

According to the International Olive Council, using olive waste in sheep diets has both economic and environmental importance, especially in regions where olive oil production is intense, such as Spain, Italy, Greece and Portugal. In Brazil, the southern region has become the main olive‐growing area, with about 20,000 ha expected to be cultivated (Saueressig et al. 2019). The same region also supports a large part of the national sheep population. The state of Rio Grande do Sul alone holds roughly 27% of all animals in the country (Magalhães et al. 2020). These conditions create a promising setting for the use of olive waste in local sheep farming. When combined with genetic improvement, modern management practices, and a stable supply of this by‐product, the approach could bring meaningful economic and environmental gains.

Different approaches have been described for adding olive by‐products to ruminant diets, helping producers lower feed costs without harming animal performance (Al Jassim et al. 1997). Studies with adult sheep suggest that olive waste can be included at up to 20% of the diet's dry matter without negative effects on digestion or productivity (Tzamaloukas et al. 2021). However, information about its influence on the digestive physiology of young lambs is still limited, particularly in relation to intestinal structure and functional responses. A detailed understanding of the digestive tract is essential to assess how such diets may affect young animals. Some anatomical and physiological features in lambs, including the reticulum, reticular groove, omasum, and intestine, remain poorly studied. In the small ruminants, the intestine, particularly the ascending colon, the *Ansa spiralis coli* (also known as the spiral ansa, spiral colon) forms a discoid spiral composed of two to four centripetal gyri that transition at the central flexure into two to four centrifugal gyri (König and Liebich 2020; Singh 2018). However, deviations from this classical pattern have been observed. In a recent study, Duro et al. (2025) reported variations in 52 out of 555 large intestines examined (9.37%), providing details on multiple types of *Ansa spiralis coli* configurations compared to the standard form (Meadows and Smith 1956; Smith 1955, 1958). Such variability highlights the importance of understanding intestinal morphology when evaluating the effects of dietary interventions provided to animals of different ages and rearing systems.

In domestic ruminants, the way alternative feeds are processed depends on the interaction between the rumen and the large intestine, both of which contribute to the final nutrient supply. Although the large intestine is often regarded as simple in form, its functional importance is greater than once assumed (Van Soest 1996). It carries out microbial fermentation, absorbs fermentation products, reabsorbs water, and shapes the faeces. In domestic ruminants, its contents may reach up to one‐fifth of the rumen volume, suggesting a notable fermentative capacity responsible for roughly 27% of cellulose and 40% of hemicellulose digestion each day (Hoover 1978). Feeding regimes also influence the architecture of the intestinal wall, as observed in cattle, goats, sheep, and camels fed diverse diets (Bello and Danmaigoro 2019; Mohamed et al. 2018; Wille 2001). Yet little is known about such effects in feedlot lambs.

Diet composition can alter the architecture of the digestive tract, especially in confined systems al tract, especially in feedlot systems (Böck et al. 2023). The limited understanding of how agro‐industrial residues act on intestinal morphology makes it difficult to develop consistent, sustainable feeding strategies (Al‐samawy et al. 2018). Although animal welfare has become a central concern in livestock production, morphological evidence connecting intestinal health with diets based on these by‐products is still scarce. Thus, in this research, we aimed to examine the effects of diets containing different proportions of olive waste on the morphology, morphometry, and wall thickness of the ascending colon in feedlot lambs. We focused on the A*nsa spiralis coli* because, as part of the large intestine, it may have greater absorptive capacity per unit volume than the rumen due to its coiled morphology, narrow shape, and slower digesta transit, which together create a fermentation reservoir facilitating cellulose metabolization, volatile fatty acid and water absorption, and faecal pellet formation. Understanding the morphological flexibility of the gastrointestinal tract is essential for interpreting how animals adapt to new feed sources. Such information can support more sustainable and efficient feeding practices in modern sheep production.

## 2 Material and Methods

The terms used to describe the results are in accordance with *Nomina anatomica veterinaria* and *Nomina histologica veterinaria* (International Committee on Veterinary Gross Anatomical Nomenclature 2017; International Committee on Veterinary Histological Nomenclature 2017).

### 2.1 Lambs and Diets

Twenty‐five male, uncastrated Ile‐de‐France X Texel crossbred lambs weaned at 55 days of age were randomly assigned to five groups (5 animals per group). All the animals were clinically healthy and received an anthelmintic therapy (Closantel, 10 g—1 mL/kg). The study was conducted in Santa Maria, in the state of Rio Grande do Sul, Brazil (29°41′29″ south latitude, 35°48′3″ west longitude and 139 m elevation). During the experimental period (September to December/2021), the average air temperature was 21.2°C and the relative humidity averaged 68%. All the lambs were kept in the same conditions, in individual stalls with slatted floors suspended 1 m off the ground, with access to mineral salt and fresh water A*d libitum*. After 10 days of adaptation to the feedlot conditions, they were weighed individually on a calibrated precision scale to record their initial live weight. All the animals were weighed every 14 days to assess weight gain until each lamb reached 36 kg live weight.

The experimental diets were then introduced according to the sample group: Group 01/control (50% corn silage); Group 02 (7.5% olive residue/42.5% corn silage); Group 03 (15% olive residue/35% corn silage); Group 04 (22.5% olive residue/27.5% corn silage); and Group 05 (30% olive residue/20% corn silage) (Table 1). The diets were formulated in ISO‐CP (18.81% crude protein) and ISO‐NDf (28% neutral detergent fibre from forage), based on the nutritional requirements of lambs for a gain of 200 g/day according to the Nutrient Requirements of small ruminants (Committee on Nutrient Requirements of Small Ruminants 2007). The relative amount of nutrients in each treatment is shown in Table 2. The diets were fed twice a day and adjusted for a 15% residue.

**TABLE 1:** Ingredients used in the formulation of the experimental diets.

Ingredients (%)	Group 01	Group 02	Group 03	Group 04	Group 05
Corn silage	50.00	42.50	35.00	27.50	20.00
Olive waste	0	7.50	15.00	22.50	30.00
Ground corn	19.85	20.11	20.36	20.36	20.89
Soybean meal	28.13	27.82	27.51	27.20	26.89
Calcitic limestone	1.02	1.07	1.13	1.17	1.22
Salt	1.0	1.0	1.0	1.0	1.0

**TABLE 2:** Levels of nutrients in the experimental diets.

Contents of DM (%)	Group 01	Group 02	Group 03	Group 04	Group 05
Dry matter	59.42	59.58	59.94	60.21	60.47
Organic matter	92.78	92.74	92.69	92.66	92.62
Crude protein	18.81	18.81	18.81	18.81	18.81
Ethereal extract	2.29	4.02	5.73	7.49	9.23
Neutral detergent fibre	26.89	27.7	28.52	29.34	30.16
Acid detergent fibre	13.42	14.67	15.93	17.19	18.44
Total carbohydrates	71.68	69.9	68.12	66.36	64.58
Total digestible nutrients	69.87	71.35	73.82	76.3	78.78
Ash	7.22	7.26	7.31	7.34	7.38
Calcium	0.60	0.66	0.72	0.78	0.84
Phosphor	0.30	0.33	0.36	0.39	0.42

*Note:* According to Valadares Filho et al. (2015).

### 2.2 Sample Collection and Biometrical Data

After evisceration of the Gastrointestinal tract (GIT) of all slaughtered lambs, the ascending colon segments were immediately immersed in cold water (5°C), firstly to check for the regularity of the *Ansa spiralis coli* (considered normal or regular when the *Gyri centripetales* (*Gcp*) start to coil from the proximal ansa to the *Flexura centralis* (*Fc*) without deviation and are commonly associated with *Gyri centrifugales* (*Gcf*) up to the start point from the *Ansa proximalis coli* (*Apc*), and all these parts resemble a disc in a single sagittal plane), and then to remove the contents by gently massaging the intestinal loops. Additionally, an aqueous solution of 4% buffered formaldehyde was injected until the intestinal lumen was filled.

The *Asc* is composed of two parts: centripetal and centrifugal gyri, determined based on anatomical textbooks and scientific literature (Constantinescu 2018; Duro et al. 2025; Habel 1985; Smith 1955). The measured length (cm) of the *Gcp* was taken from the starting point of the *Apc* to the *Fc* in the point that the *Apc* meets the last *Gcf* in the most craniodorsal point of the spiral disc, and the length of the *Gcf* was measured from the *Fc* to its terminal part in the distal ansa, which corresponds almost to the same point as the start of the *Gcp*. The measurements were made using a body tape measure.

### 2.3 Light Microscopy

**FIGURE 1:**
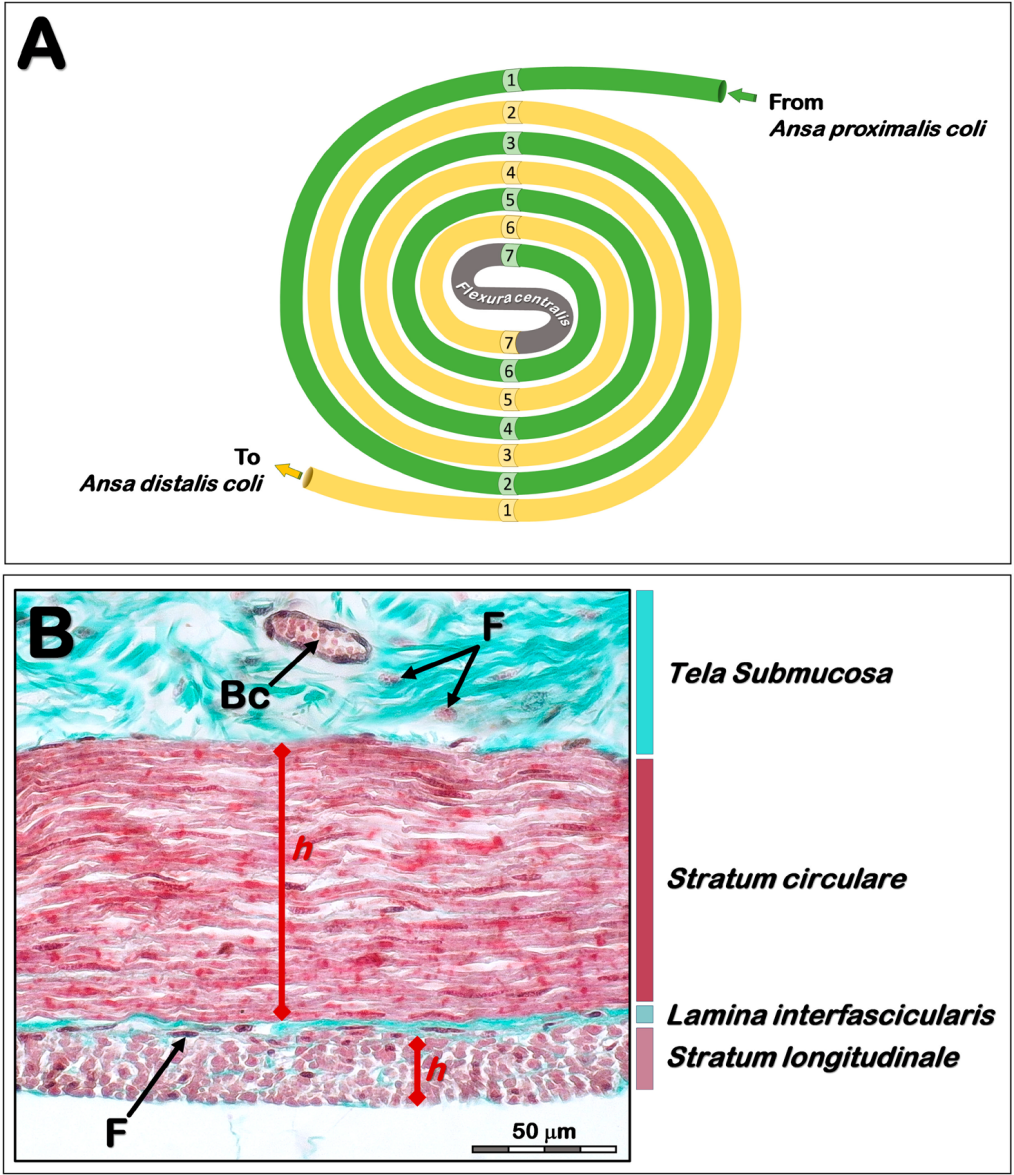
(A) Schematic representation of *Ansa spiralis coli* indicating the sample collection sites for microscopic analysis. (B) Light photomicrograph (Masson‐Goldner trichrome) of the ascending colon in lambs fed olive wastes: Cross‐section of the wall of the *Ansa spiralis coli* showing the *Stratum circulare* and *Stratum longitudinale* of the *Tunica muscularis*. Bc, blood capillaries; F, fibroblasts.

After fixation, circular sections (5 mm) carefully oriented in a vertical direction were collected in every centripetal and centrifugal loops according to Figure 1A. After washing with PBS, the samples were dehydrated in increasing concentrations of alcohol (70%–100%), diaphanized in xylene and embedded in histological paraffin. The blocks were cut to a thickness of 6 μm using a semi‐automatic microtome (RM‐2065, Leica, Wetzlar, Germany) and the slides were stained with Masson‐Goldner trichrome.

### 2.4 Digital and Statistical Analysis

Four hundred and fifty slides were used to calculate the thicknesses of the mucosal and muscular tunics of the centripetal and centrifugal loops. Five images of random fields from each loop were captured using a light microscope (Zeiss AX10 Scope A1) coupled with a digital camera (Zeiss Axiocam 105 colour) with a 10× objective lens.

To calculate the total thickness of the *Tunica mucosa* (*TMu*), up to three perpendicular lines were drawn between the highest *adluminal* point of the epithelium and the *Lamina muscularis mucosae* in each image. The thicknesses of the sublayers of the *Tunica muscularis* (*TMs*) were calculated using the same methodology. However, for the *Stratum circulare*, the vectors were drawn between the lowest points of the submucosal layer and the *Lamina interfascicularis*. For the *Stratum longitudinale*, the vectors were drawn from the *Lamina interfascicularis* to the border with the *Tunica serosa* (Figure 1B).

The relative content (%) of the main components of the *TMu* was calculated by automatic particle selection based on colour difference in relation to the total area of the sample in the background of the image. All images were randomized and analysed in a double‐blind manner using Image J software version 1.51 J—Fiji. (Rueden et al. 2017).

Statistical analysis was performed using SAS (Statistical Analysis System) Software version 9.4 MB (SAS Institute Inc. 2023). The normality of all variables was verified by the Shapiro–Wilk test, followed by the One‐way ANOVA test (*p* < 0.05) and comparison between means by the Tukey test (*p* < 0.05).

## 3 Results

### 3.1 Macroscopic Aspects

In all analysed samples of the *Asc*, which normally should be spiral discoid, it was found that the hemispherical spiral shape was predominant and no discoid was found. Our attention in this study was only to note in general the shape of the *Asc*, the number of centripetal and centrifugal loops and their respective lengths and not to note in detail whether there were any deviations from the normal spiral shape. From the careful observations of all large intestine samples, it was recorded that all specimens had 3 *Gcp* and 3.5 *Gcf* and the lengths of the *Gcp* and *Gcf* were measured separately. These two records were important objectives of this study. The total mean length of the *Gcp* was 102.3 ± 14.24 cm, while that of the *Gcf* was 86.5 ± 12.87 cm. The total mean values for each group were compiled in Table 3.

**TABLE 3:** *Ansa spiralis coli* measurements in lambs fed different concentrations of olive waste.

		Length (cm)		Thickness (μm)					
				Tunica mucosae		Tunica muscularis			
						Stratum circulare		Stratum longitudinale	
		Mean–SD	Min–Max	Mean–SD	Min–Max	Mean–SD	Min–Max	Mean–SD	Min–Max
*Gyri centripetales*	Group 1	96.7 ± 19.13	121.5–80.8	378.43 ± 51.84	317.89–438.47	112.38 ± 41.05	77.09–162.50	39.39^a^ ± 10.84	77.1–163
	Group 2	106.0 ± 14.53	121.0–87.0	383.68 ± 46.89	311.42–430.76	90.56 ± 9.09	82.21–105.38	37.94^a^ ± 4.02	82.9–105
	Group 3	100.3 ± 13.54	117.4–82.5	453.08 ± 64.16	335.98–530.98	107.70 ± 14.37	89.84–127.52	47.25^a^ ± 6.40	88.7–137
	Group 4	101.6 ± 12.49	114.5–84.5	379.59 ± 57.88	298.1–428.9	92.80 ± 19.61	76.49–121.32	41.15^a^ ± 4.68	76.5–121
	Group 5	106.0 ± 16.19	128.4–83.1	408.69 ± 65.61	312.44–485.8	112.36 ± 31.24	79.99–154.51	52.50^b^ ± 11.61	71.8–149
	Mean/SD	102.3 ± 14.24		400.3 ± 31.78		103.16 ± 27.54		43.65 ± 9.79	
	Pr>F	0.8641		0.034		0.495		0.670	
	SE	0.0623		0.364		0.137		0.098	
*Gyri centrifugales*	Group 1	81.8 ± 16.38	96.5–63.0	354.55 ± 81.95	285.2–450.44	115.68 ± 38.58	77.09–162.50	39.01 a ± 8.46	84.5–161
	Group 2	89.2 ± 16.6	104.5–61.5	315.64 ± 53.59	262.17–404.5	90.21 ± 8.57	82.21–105.38	35.80a ± 2.09	80.2–99.5
	Group 3	86.2 ± 8.72	98.0–72.2	386.03 ± 41.92	330.72–443.1	102.32 ± 11.38	89.84–127.52	46.79a ± 6.51	87.7–125
	Group 4	83.4 ± 15.12	95.3–62.5	323.61 ± 42.52	263.86–364.45	89.67 ± 13.95	76.49–121.32	40.14a ± 4.38	72.1–106
	Group 5	90.4 ± 12.2	107.5–75.0	338.18 ± 71.06	246.43–439.26	110.58 ± 35.40	79.99–154.51	53.31b ± 11.44	59.6–142
	Mean/SD	86.5 ± 12.87		343.60 ± 65.32		101.69 ± 25.06		43.01 ± 9.64	
	Pr>F	0.8626		0.014		0.433		0.602	
	SE	0.0627		0.422		0.153		0.112	

*Note:* Mean ± SD (min max); ^a,b^Significantly different (*p* < 0.05); one‐way ANOVA.

**FIGURE 2:**
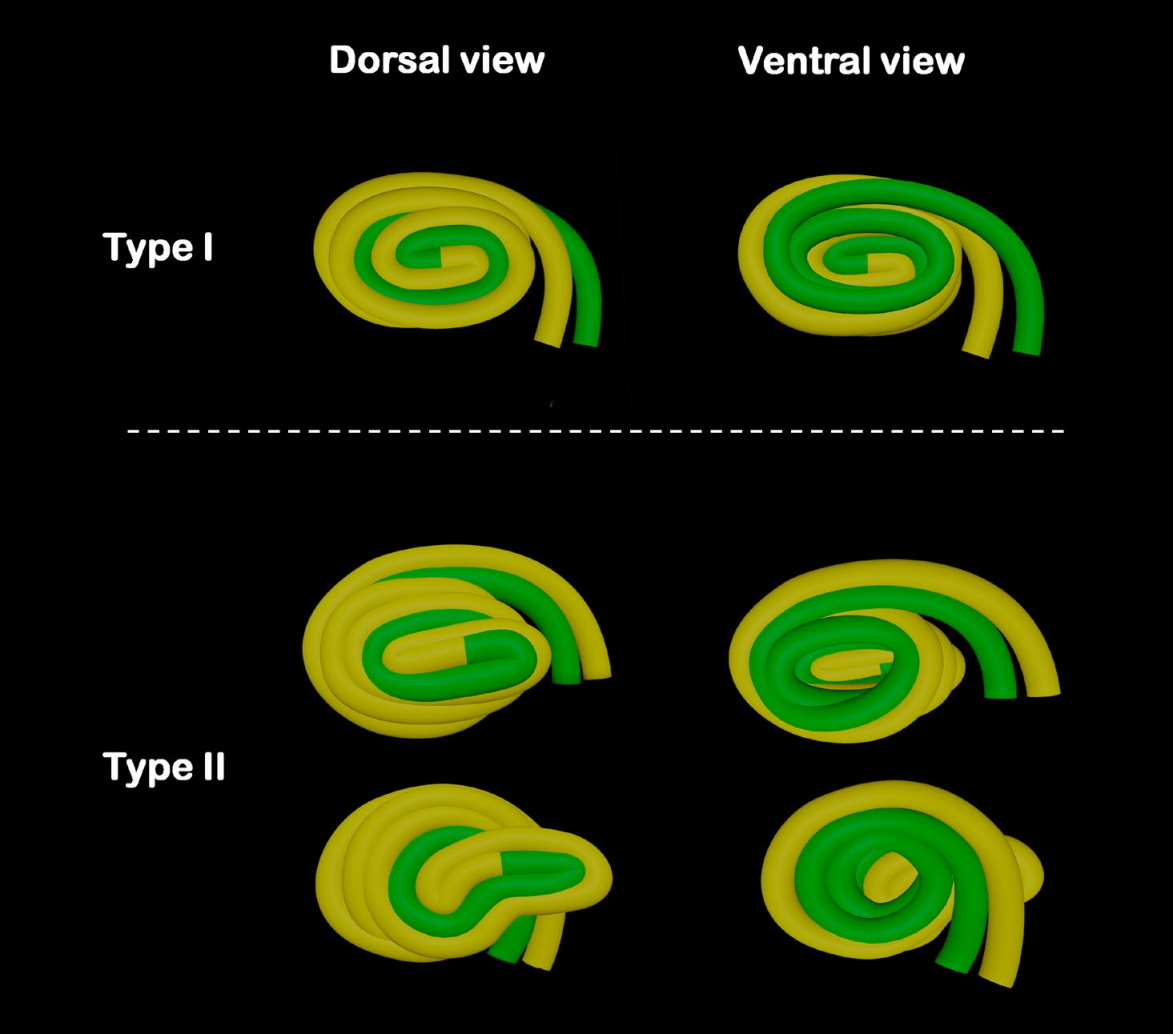
Three‐dimensional diagram of *Ansa spiralis coli* of lambs showing the topographical arrangement of centripetal (yellow) and centrifugal (green) loops in Type I and Type II patterns.

However, the spatial arrangement of these segments varied among specimens. Two distinct patterns were identified. In the ‘Type 1’ pattern, observed in 43.9% of samples, the loops formed a double spiral with the *Fc* positioned at the center. In this configuration, the *Gcp* were located externally and the *Gcf* internally, giving the structure a slightly conical appearance. In the ‘Type 2’ pattern (56.1%), the final *Gcp* showed an expansion that extended beyond the outermost portion of the centripetal loop (Figure 2).

### 3.2 Microscopic Aspects

**FIGURE 3:**
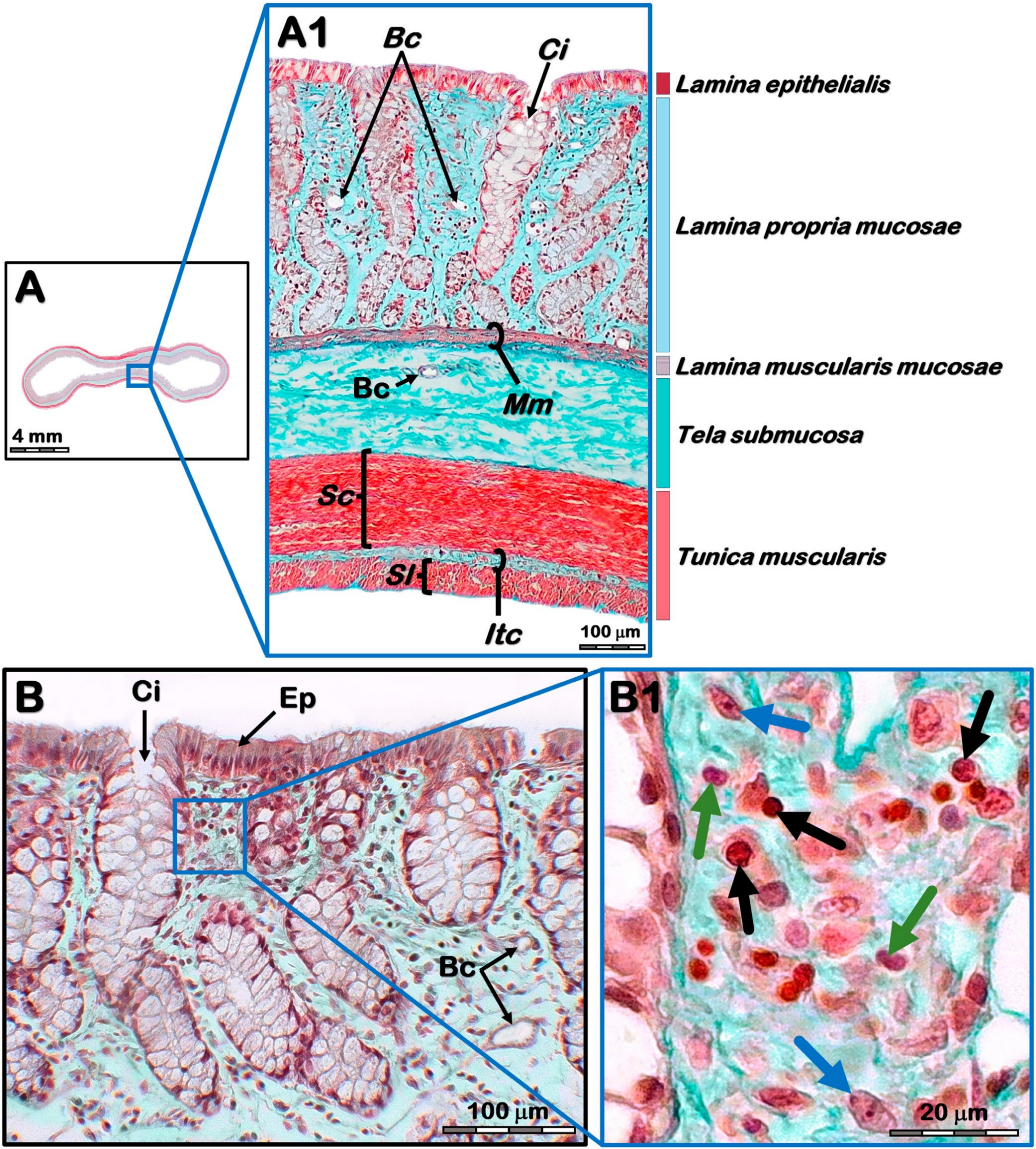
(A) Light photomicrograph (Masson‐Goldner trichrome) of the ascending colon in lambs fed olive wastes: (A1) Cross‐section of the wall of the *Ansa spiralis coli*: Bc, blood capillaries; Ci, *crypta intestinalis*; Mm, *LAMINA muscularis mucosae*; Sc, *stratum circulare* of the *Tunica muscularis*; Sl, *Stratum longitudinale* of the *Tunica muscularis*; Itc, Interfascicular connective tissue. (B) *Lamina propria mucosae* at high magnification: Ep, *epithelium simplex columnare*. (B1) Black arrows, lymphocytes; Green arrows, plasmocytes; Blue arrows, fibroblasts.

Using Masson Goldner trichrome staining, it was possible to distinguish the *TMu*, *submucosal layer* (*TSm*) and *TMs* (Figure 3A). The *Tunica serosa* was not included in the analyses because it was occasionally lost during sample processing. The *TMu* consisted of three sublayers. On the adluminal side, the *Lamina epithelialis* consisted of columnar cells with a striated border and microvilli located at the apical pole. The *Lamina propria mucosae* (*LPm*) consisted of loose connective tissue associated with non‐encapsulated (or diffuse) lymphoid tissue and a dense rete of blood capillaries. The epithelium continued into the *LPm*, characterizing the *Crypta intestinalis*. In well‐oriented cross sections, they were directed vertically in the abluminal direction. On the abluminal side, the *Lamina muscularis mucosae* (*LMm*) was interposed between the *TMu* and the *TSm*. It consisted of two thin layers of smooth muscle oriented in the circular and longitudinal directions. The *TSm* consisted of loose connective tissue and contained vascular plexuses, nerve fibres from the submucosal plexus, and typical cellular elements.

The *TMs* consisted of two layers of smooth muscle. Internally, the *Stratum circulare* was thicker, and the smooth muscle cells were oriented in the circumferential direction. The *Stratum longitudinale*, in contrast, was noticeably thinner with fascicles of smooth muscle cells arranged in the longitudinal direction. In addition, it was possible to observe the *Lamina interfascicularis* located between the circular and longitudinal muscle layers. It consisted of sparse bundles of connective tissue and associated fibroblasts (Figure 3A1). The base of the crypts consisted of cells with well‐characterized secretory vacuoles (*Exocrinocytus caliciformis*). However, in no specimen was it possible to distinguish *Exocrinocytus cum granulis acidophilicis* or Paneth cells (Figure 3B). In addition, three types of cells were observed distributed in the *LPm*. Lymphocytes were the predominant type, but it was also possible to distinguish plasma cells and undifferentiated fibroblasts (Figure 3B1).

#### 3.2.1 Wall Thickness of *Ansa Spiralis Coli*

The absolute thickness of the *TMu* and the *TMs* of the wall of the *Asc* were analysed and recorded separately for the *Gcp* and *Gcf* and are presented in Table 3 for all groups of samples. In addition, the total thicknesses of the *Stratum circulare* and *Stratum longitudinale* of the *TMs* were considered separately.

#### 3.2.2 Tunica Mucosa Content

We analysed separately, in each group, the relative content of the main fibrocellular components (Crypta intestinalis, non‐encapsulated lymphoid tissue and connective tissue) of the TMu of the *Asc*. As the statistical analysis did not reveal differences between the groups, the results were presented as mean values per segment of the *Asc*, without distinction between groups. Thus, for the Gcp, the mean content of Crypta intestinalis was 38.81% ± 5.38%, while in the Gcf it was 39.35% ± 4.86%. For Gcp, non‐encapsulated lymphoid tissue occupied 8.54% ± 1.43% of the mucosal area, while in Gcf it occupied 8.49% ± 1.38%. In addition, the mean connective tissue content was 52.65% ± 5.13% in Gcp, while in Gcf it was 52.16% ± 4.49%. The maximum and minimum data, in addition to others, in each group were compiled in Table 4.

**TABLE 4:** *Tunica mucosa* content (%) of *Ansa spiralis coli* in lambs fed different concentrations of olive waste.

		Crypta intestinalis		Lymphoid tisue cells/blood vassels		Conective tissue	
		Mean–SD	Min–Max	Mean–SD	Min–Max	Mean–SD	Min–Max
*Gyri centripetales*	Group 1	37.13 ± 4.14	31.19 43.19	8.17 ± 1.86	4.77–11.02	54.70 ± 4.59	45.78–60.18
	Group 2	35.85 ± 6.33	27.93 49.08	9.17 ± 1.42	7.12–12.69	54.98 ± 5.73	43.66–63.10
	Group 3	40.48 ± 4.81	31.29 48.18	8.43 ± 1.28	6.17–10.04	51.10 ± 5.19	43.38–62.47
	Group 4	40.68 ± 4.45	35.52 48.69	8.73 ± 1.26	7.44–11.23	50.59 ± 3.60	43.70–55.13
	Group 5	39.95 ± 5.42	32.28 51.69	8.16 ± 1.24	6.46–10.71	51.88 ± 4.99	39.95–57.12
	Mean	38.81 ± 5.38		8.54 ± 1.43		52.65 ± 5.13	
	Pr>F	0.312		0.215		0.041	
	SE	0.058		0.072		0.118	
*Gyri centrifugales*	Group 1	38.59 ± 3.02	33.84 43.94	8.27 ± 1.42	5.94–10.28	53.14 ± 2.64	49.02–58.18
	Group 2	38.24 ± 6.71	26.81 49.25	9.09 ± 1.34	6.62–11.20	52.68 ± 5.87	44.13–63.04
	Group 3	39.97 ± 3.94	34.57 46.47	7.65 ± 1.14	5.69–9.46	52.38 ± 4.66	45.28–59.03
	Group 4	39.63 ± 4.17	31.32 44.49	9.19 ± 0.89	7.96–10.55	51.19 ± 4.14	46.11–60.30
	Group 5	40.24 ± 5.52	28.91 47.55	8.37 ± 1.54	6.17–11.67	51.39 ± 4.49	46.18–60.34
	Mean	39.35 ± 4.86		8.49 ± 1.38		52.16 ± 4.49	
	Pr>F	0.678		0.184		0.392	
	SE	0.023		0.079		0.046	

*Note:* Mean ± SD (min max); Significantly different (*p* < 0.05); one‐way ANOVA.

#### 3.2.3 The Performance of Lambs Confined

The performance of lambs of all groups in this research from the initial to the slaughterhouse is presented in the Table 5.

**TABLE 5:** Performance of lambs confined with different concentrations of olive residue.

	Diet					Pr>F	SE
	Group 01	Group 02	Group 03	Group 04	Group 05		
Initial liveweight (kg)	20.5	19.7	19.3	18.8	17.9	0.741	0.622
Live weight at slaughter (kg)	37.3	37.3	37.3	36.6	37.2	0.925	0.272
Mean daily gain (kg)	0.33	0.33	0.32	0.26	0.26	0.079	0.010
Feed conversion (kg)	4.13	3.91	3.94	4.01	3.78	0.751	0.082
Days to slaughter	55.1	56.0	60.4	69.9	74.6	0.192	3.023
Rumination time (min./day)	461.4	524.3	513.3	457.1	575.7	0.010	10.914
Eating time (min./day)	191.6	230.0	210.0	185.0	135.7	0.0002	6.046
Mastication time (min./day)	672.8	754.3	723.3	632.8	711.4	0.0616	13.156

*Note:* Means; Shapiro–Wilk Normality Test; One‐Way ANOVA (*p* < 0.05 significance).

## 4 Discussion

The morphoplastic characteristics of the large intestine, with distinct functional capacities in each segment, are indispensable conditions for ensuring good animal performance indices (Celi et al. 2017). In this sense, the ascending colon of ruminants stands out for its unique morphological aspects that play a crucial role in the digestive process. Specifically, we focus on the *Asc*, a segment that, due to its peculiar helical arrangement, reduced lumen and slower transit of digesta, also performs as a fermentation site, facilitating cellulose metabolization. In contrast to previous knowledge, the large intestine is now recognized as a segment of critical morphofunctional relevance. Its functions go beyond the formation of faeces, encompassing microbial fermentation of remaining substrates, absorption of short‐chain fatty acids (SCFAs), and reabsorption of water and electrolytes (Dixon and Nolan 1982). According to Wang et al. (2019) and Lima et al. (2019), the ascending colon acts effectively in the recovery of metabolizable energy, a vital component of energy homeostasis and overall metabolism in small ruminants. It also stands out for its high efficiency in fibre degradation, which is essential for adaptation to fibre‐rich diets, such as those evaluated in this study. This information can support more sustainable and efficient feeding practices in modern sheep production.

The *Asc* is a segment of the *Colon ascendens* with distinct morphological attributes that plays a crucial role in the digestion and performance of ruminants. It has a complex helical arrangement where the initial segment is made up of the *Gcp* that spiral up to the *Fc*. From this point, the *Gcf* continue in the opposite direction, lodging precisely in the interspaces of the centripetal loop. Although we recognize that the topographical evaluation of the visceral complex *Ex situ* represents a limitation of this study, this did not compromise the precise identification of all segments of the *Asc*. When observed in isolation outside the abdominal cavity, the *Asc*, which usually resembles a spiral discoid, in fact all analysed samples were hemispherical and the *Gcp* were situated somehow dorsally while the *Gcf* occupied the ventral position. Thus, the double helical shape cannot be spread out completely on a flat surface, taking on a discoidal shape as is usually described in animal anatomy textbooks (König and Liebich 2020; Nickel et al. 1979; Singh 2018). With regard to this, it is admissible to consider that during prenatal development, the loops are twisted, assuming a snail shell appearance, with the *Fc* situated at the most dorsal point, as reported by Kolda (1931) or as it was described by Habel (1985), or in the study of Rajput (2006), which confirmed that in Gaddi sheep, the shape of the entire *Ansa spiralis coli* resembles a hemisphere.

The results of this study, observed in Texel/Ile‐de‐France crossbred lambs, demonstrated a notable morphological consistency in relation to the amount of *Gcp* (3) and *Gcf* (3.5). These data are highly similar to those observed by Duro et al. (2025) in adult Bardhoka sheep, which had an average of 3.25 loops of *Gcp* and 3.5 of *Gcf*, respectively. Regarding *Asc* dimensions, the data in Table 3 reveal that the mean length of *Gcp* was slightly greater than that of *Gcf*, resulting in a ratio of 1.18 cm between them. These data also corroborate the reports of Duro (1.22 cm) in adult sheep. We recognize that the minimal differences observed are associated with variables such as the age and breed of the animals in the respective studies. Thus, the morphological differences of the ascending colon reflect specific adaptations determined by breed according to age, associated with the processing of increasing volumes of dry matter. According to Demment and Van Soest (1985), the intestinal capacity of ruminant herbivores increases linearly with body weight, from which a ratio can be deduced: metabolic requirement/intestinal capacity (MR/IC). Since the retention time of food particles in the intestine is proportional to this ratio, larger animals are able to retain digesta for longer, which favours more complete digestion. We therefore agree that the lower MR/GC ratio observed in medium‐sized herbivores, such as sheep, exerted selective pressure for the development of morphological adaptations in the gastrointestinal tract that selectively slow the passage of digesta, increasing digestive efficiency. Although we know that intestinal passage speed is also influenced by the rumination process—which in turn is directly related to particle size (Ueda et al. 2001)‐rumination is only energetically advantageous within a limited range of body sizes. Thus, small ruminants are characterized by maximum rumen fermentation rates and depend almost exclusively on microbial production of short‐chain fatty acids (SCFAs) as an energy source, supplemented by post‐ruminal intestinal fermentation to obtain other essential nutrients.

However, in addition to these minor macroscopic variations, both segments of the ascending colon had similar microscopic characteristics, with histological organization adapted to the formation of faeces and the absorption of vitamins, water, and electrolytes (Banks 1993). In all groups in this study, the tissue architecture of the *Asc* wall was similar to other ruminant species, with emphasis on the presence of longer and straighter intestinal glands, with a specific number of goblet cells as previously reported by Eurell and Frappier (2006) and König and Liebich (2020). In sheep, the *TMu* was the innermost and most functional layer, responsible for direct interaction with the luminal contents. Previous studies have reported that the mucosa of the ovine colon is composed of simple prismatic epithelium in which absorptive cells, reserve cells and enteroendocrine cells predominate, as well as *Exocrinocytus caliciformis* which secrete mucus to protect the mucosa and facilitate the transit of intestinal contents (Banks 1993; Ham and Cormack 1983).

It is known that the epithelium of the colonic mucosa acts as a barrier between internal and external environments, but also regulates nutrient absorption and waste secretion through selective permeability (Turner 2009). In addition, the mucosa is also where the associated lymphoid tissue interacts with the external environment. The magnitude of this interaction is most intense in the *Asc*, which is the largest mucosal surface that is in continuous contact with food antigens and various microorganisms. This dual function positions the *TMu* epithelium as a mediator of interactions between the mucosa‐associated immune system and components of the intestinal lumen, such as food antigens and microbial metabolites. In addition, most intestinal mucosal surfaces are covered by a hydrated gel formed by mucins that are secreted by intestinal goblet cells. In the samples analysed in this study, the significant presence of these cells suggests an efficient defence system, since the mucous gel acts as a physical barrier, limiting the direct contact of particles and microorganisms with the intestinal epithelium, as described by Johansson et al. (2008). Although small molecules can easily pass through the mucus layer, mass flow is restricted, favouring the formation of an unstirred layer of fluid adjacent to the epithelium. However, in the large intestine, this layer can slow down the absorption of nutrients by reducing the rate of diffusion to the microvilli, where the carrier proteins are concentrated. Although defects in this layer were not specifically assessed in this study, the lower performance of lambs in Group 5 suggests a possible association with this variable. In addition, the chemical composition of the olive residues used in this study, with approximately 30% NDF in this group, probably contributed to the effects observed. Thus, the presence of insoluble fibres may have stimulated greater chewing and rumination time (Table 5), increasing intestinal motility in group 5, resulting in greater thickness of the *TMs*, while in the other groups with up to 22.5% inclusion, there were no significant changes in this parameter.

Olive waste has been the focus of studies exploring its potential as a feed in animal farming, especially for ruminants (Chiofalo et al. 2004; Martín García et al. 2003; Molina‐Alcaide and Yáñez‐Ruiz 2008). It includes significant concentrations of bioactive substances such as polyphenols (hydroxytyrosol, oleocanthal), monounsaturated fatty acids (oleic acid) and insoluble dietary fibre, which promote intestinal health by acting as antioxidants, antimicrobial agents and prebiotics (Ribeiro et al. 2021). Although our main objective is to evaluate the effects of olive waste on the morphometry of the *Asc*, it is important to note that both olive oil and waste contain bioactive substances with protective effects on the colon. Studies have already shown that olive oil modulates the inflammatory pathway in colitis and inhibits tumour growth in murine models (Nanda et al. 2018; Sánchez‐Fidalgo et al. 2013), In this context, it is possible to conclude that the polyphenols and fatty acids in olive waste can also reduce inflammation and cancer cell proliferation. Our data showed that the different inclusion levels of olive waste did not affect the content of intestinal inflammatory lymphoid cells, supporting its possible role in protecting the intestinal mucosa by modulating the microbiota and producing short‐chain fatty acids. However, given the complexity of the mechanisms involved, more studies are required to validate these hypotheses and optimize the application of olive waste in animal nutrition.

The gastrointestinal tract is highly sensitive to variations in the macronutrient composition of the diet (Park et al. 2013). Current studies have shown that the ability of the gastrointestinal tract to detect dietary fats affects intestinal functions, including secretion, motility and transit speed of contents (Boyd et al. 2003; Li et al. 2011). Thus, higher levels of dietary fat can be toxic to rumen microorganisms and affect fibre digestibility, resulting in reduced feed intake and animal performance (Behan et al. 2019). We observed significant behavioural changes in the animals in confinement, with the specimens in group 5 showing shorter feeding times and longer rumination times. One‐way ANOVA revealed a significant difference in relative TMs thickness among the groups (Table 3). Thus, the effect size (*η* ^2^ = 0.098) indicates that only 9.8% of the total variance in muscle thickness was explained by the experimental condition, suggesting that the practical effect of the treatments is modest. When analysing the data as a whole, the results suggest a possible association between lower feed conversion ratios and the lower average daily weight gain observed in group 5, which may be related to changes in gastrointestinal motility, as indicated by the greater thickness of the *TMs* observed in the specimens from that group.

According to Patten et al. (2015), the mechanism by which palm oil supplementation increases rat intestinal contractility in vivo may involve the activation of gastrointestinal agonists or specific ion channel receptors that regulate calcium flow within the smooth muscle cells of the ileum and colon. In addition, some authors have shown that acute administration of polyunsaturated fatty acids not only affects various ion channels, including the L‐type calcium current of the cell sarcolemma (Pepe et al. 1994; Xiao et al. 1997), but also directly affects the calcium content of the sarcoplasmic reticulum (Negretti et al. 2000). Thus, we hypothesize that the increased contractility of *Asc* smooth muscle cells in lambs in group 5 may involve similar ion flow mechanisms. These factors may have modulated the greater *TMs* thickness. Dietary supplementation with polyunsaturated fatty acids also affected calcium signalling in rat T‐cells (Triboulot et al. 2001). However, we found no further details of this interaction with intestinal smooth muscle. As the large intestine is primarily responsible for dehydrating and forming stool, it is possible to hypothesize that the contractile properties influenced by polyunsaturated fatty acids in the ileum may be transferred to the colon. In this context, future studies addressing the binding of gastrointestinal agonist receptors, complemented by reverse transcription polymerase chain reaction technologies, may contribute to a better understanding of how the effects of high levels of fatty acids in the diet increase the contractility of the *TMs* in ruminants. In addition, it is known that reducing the pH in the colon promotes an increase in passage speed in the large intestine, depending on the composition of the diet and the animal model used (Glitsø et al. 1998). Moreover, short‐chain fatty acids are implicated in the control of gastrointestinal contractility and motility in the small intestine (Cuche 1999) and colon (Cherbut et al. 1998). In light of this, future studies demonstrating the interaction between intestinal pH and the content of short‐chain fatty acids involved in intestinal contractility/motility may expand our knowledge of the effects of diets containing more than 30% olive waste on TMs thickness.

According to the Food and Agriculture Organization of the United Nations, the consumption of animal protein by an expanding human population is expected to increase by more than 50% over the next four decades (Dijkstra et al. 2013). As a result of this population growth, it is essential to improve efficiency in the use of food resources in intensive animal production systems. In addition, the wider diversity of feed sources derived from regional agro‐industrial waste reduces water consumption and the occupation of arable land, fostering an economically viable and environmentally efficient production chain. Feeding animals with agro‐industrial by‐products is an old practice. Among the main advantages is less dependence on traditional roughage and a reduction in costs related to waste management (Malenica et al. 2022; R. Singh et al. 2021). Our results highlight that the inclusion of up to 22.5% olive residues in feed for confined lambs can be a viable strategy for producers, especially in regions with high availability of olive residues, such as southern Brazil and Mediterranean countries. The more efficient use of local alternative dietary ingredients can alleviate the current environmental pressure on the global livestock sector by promoting a more sustainable production chain.

This research demonstrated that the inclusion of up to 22.5% olive waste in the diet can partially replace traditional roughage, reducing costs and the environmental impact in olive oil‐producing regions. In addition, the fibrocellular content of the *Ansa spiralis coli* wall remained unchanged between the groups. These results are consistent with the general benefits of olive oil, confirming its role in the intestinal health of confined animals. In contrast, diets containing 30% olive residue resulted in a significant increase in the thickness of *Tunica muscularis*, affecting intestinal contractility and negatively impacting animal performance.

## Funding

This study did not receive any specific grant from funding agencies in the public, commercial, or not‐for‐profit sectors.

## Ethics Statement

This study was approved by the Honorary Ethical and Experimental Animals Committee of the Federal University of Santa Maria (CEUA/UFSM n° 8,088,120,419).

## Conflicts of Interest

The authors declare no conflicts of interest.

## Data Availability

The data that support the findings of this study are available on request from the corresponding author. The data are not publicly available due to privacy or ethical restrictions.
